# Enigmatic Fossils from the Lower Carboniferous Shrimp Bed, Granton, Scotland

**DOI:** 10.1371/journal.pone.0144220

**Published:** 2015-12-23

**Authors:** Mikołaj K. Zapalski, Euan N. K. Clarkson

**Affiliations:** 1 Faculty of Geology, University of Warsaw, Żwirki i Wigury 93, 02–089, Warszawa, Poland; 2 School of Geosciences, University of Edinburgh, West Mains Road, Edinburgh, EH9 3JW, United Kingdom; UC Irvine, UNITED STATES

## Abstract

The Lower Carboniferous (Visean) Granton Lagerstätte (Edinburgh, Scotland) is principally known for the discovery of the conodont animal, but has also yielded numerous crustaceans and other faunas. Here we report on small branching colonies, reaching 10 mm in length. They are small, erect, arborescent, and irregularly branched with predominant monopodial and dichotomous growth. They bud in a single plane. In one specimen the wall microstructure is well preserved and it is composed of evenly spaced, linear fibers, running parallel to the axis of the stems, and connected by transverse bars. We discuss possible biological affinities of these organisms; we consider algal, poriferan, hydrozoan and bryozoan affinities. The general pattern of branching, presence of fan-like structures (interpreted here as possible gonophores) and microstructure suggests affinity to Hydrozoa, affinity to non-calcifying algae is less likely. Assuming hydrozoan nature; the microstructure might suggest affinities with the extant family Solanderiidae Marshall, 1892 that possess an internal chitinous skeleton. The EDS analysis shows that fossils discussed here are preserved as phosphates. The skeletons were probably not mineralized, the presence of phosphorus suggests that the colonies were originally composed of chitin. We describe these organisms as *Caledonicratis caridum* gen. et sp. nov. (Solanderiidae?, Capitata?). Colonies of *C*. *caridum* gen et. sp. nov. sometimes encrust the exuviae of crustaceans, which very probably lived in fresh to brackish water thus indicating a likely habitat of *Caledonicratis*.

## Introduction

**Fig 1:**
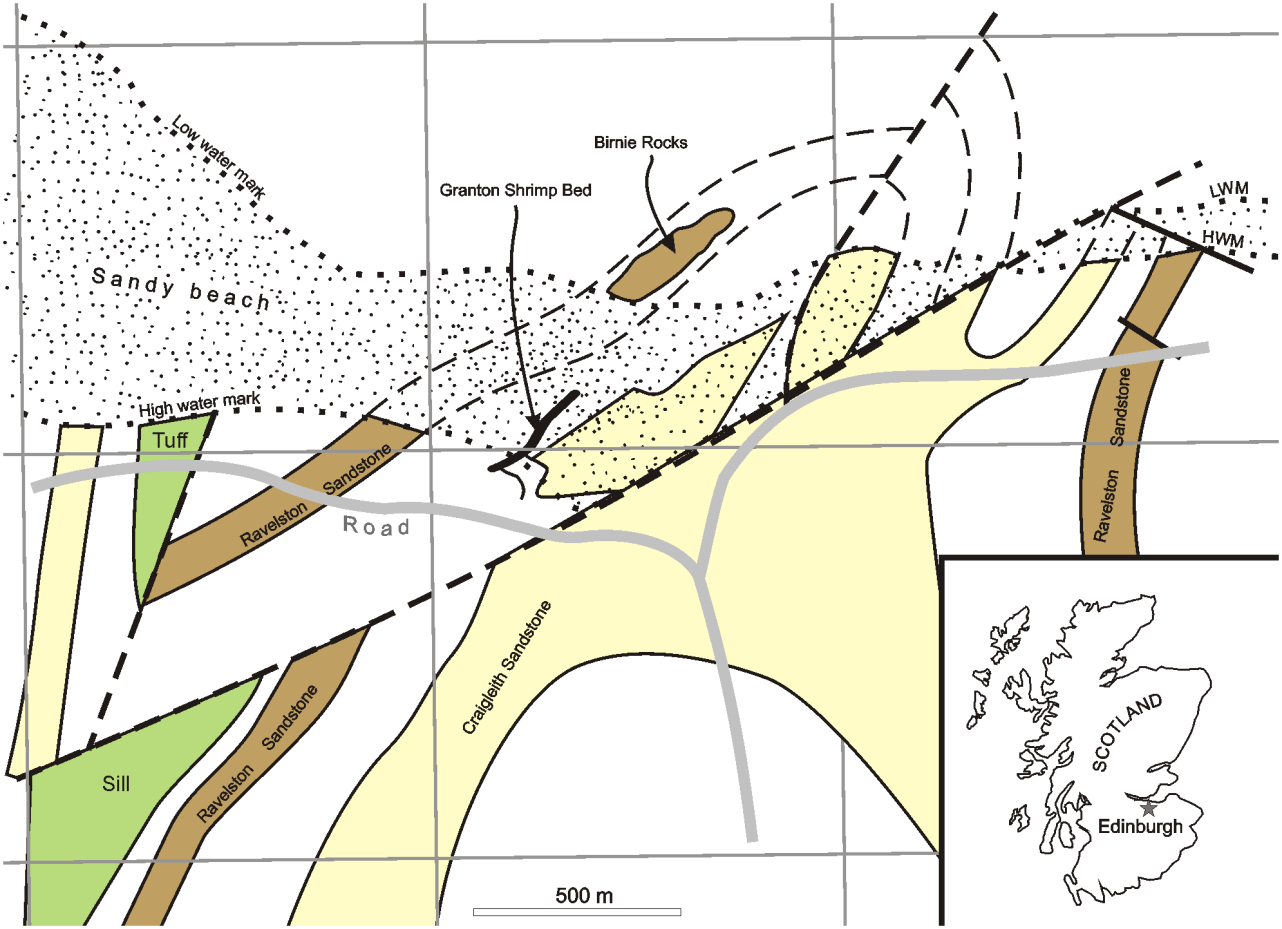
A map showing the locality of the Granton Shrimp Bed at Granton, Edinburgh.

The Granton Lagerstätte (near Edinburgh, Scotland; Fig 1) is principally known for the discovery of the conodont animal [1], but has also yielded numerous crustaceans (e. g., [2, 3, 4]) and other faunas (e.g., [5, 6]). Among vertebrates, molluscs and various invertebrates, Briggs and Clarkson [5] described a certain number of branching fossils that they considered as either bryozoans, hydrozoans, or algae. The aim of this paper is to describe them in detail and to discuss their possible hydrozoan affinities.

LWM and HWM refer to Low Water Mark and High Water Mark respectively. Based upon British Geological Survey geological map. 1:50000 Series. Edinburgh Scotland Sheet 32 E [7], with the permission of the British Geological Survey.

Cnidarians, a phylum close to the root of the evolutionary tree of animals [8], are common fossils, but not all groups are represented evenly throughout time. Corals of various groups, due to the presence of a carbonate skeleton have a good fossil record; on the other hand, hydrozoans, being usually soft-bodied, are quite rare. Therefore hydrozoans, despite their long evolutionary history, are scarce as fossils. In contrast to their rarity in the fossil record, recent hydrozoans are very diversified, with more than 3,500 recent species known [9], as compared to corals with about 6,000 species known. As a result of such a great diversity hydrozoan phylogeny has been intensively studied [10, 11, 12]. The fossil record provides a necessary tool for the calibration of molecular phylogenetic trees and for that reason every discovery of a fossil hydrozoan is important for the understanding of evolutionary history of this group.

Several soft-bodied taxa of hydrozoans have been described from the Palaeozoic; the oldest hydrozoan medusae are Cambrian [13] and the oldest hydrozoan polyps are these of *Sinobryon elongatum* Baliński et al. 2013 from the Ordovician of Hubei, China [14]. A limited number of other non-skeletal hydroids has been reported throughout the Palaeozoic, but most of them, due to a poor state of preservation were described under open nomenclature [15]. In some cases, however, it has been possible to assign fossil hydroids to modern families. The porpitoid *Pseudodiscophyllum vindermerense* Fryer & Stanley 2004 (incorrectly ‘*vindermerensis*’ in the referred publication) has been described from the Silurian of Cumbria, England [16]. Several plumularioid species of the genus *Plumalina* Hall 1858 from the Silurian and Devonian of North America have been reviewed recently [15].

The exceptional preservation of one of the specimens discussed here might suggest their possible affinities with the recent family Solanderiidae Marshall, 1892; the impact of this new discovery for the calibration of molecular-based trees is discussed.

## Material and Methods

The material consists of 36 specimens. The collection is depostited at the National Museums of Scotland, Edinburgh, the repository numbers starting with NMS and RSM are given in the description section with the holotype and paratypes. Specimens were studied under a Zeiss Discovery.V20 stereoscopic microscope, using both polarized and unpolarized light at various angles, at magnifications from 10× to 150×, and SEM (the microstructure images were taken in 1980's at the University of Edinburgh; others were taken at the Faculty of Biology, University of Warsaw). Photos were taken with a Canon EOS 70D camera either using Zeiss Discovery.V20 stereoscopic microscope or using Canon EF 100mm f/2.8L Macro IS USM Lens. Energy Dispersive Spectroscopy (EDS) analyses on three uncoated specimens were performed at the Faculty of Geology, University of Warsaw with Zeiss Sigma VP SEM, at 20kV and pressure 40 Pa. This method is commonly used in analysis of element distribution (elemental mapping) in soft-bodied fossils (see explanations and case studies in [17] and [18])

The electronic edition of this article conforms to the requirements of the amended International Code of Zoological Nomenclature, and hence the new names contained herein are available under that Code from the electronic edition of this article. This published work and the nomenclatural acts it contains have been registered in ZooBank, the online registration system for the ICZN. The ZooBank LSIDs (Life Science Identifiers) can be resolved and the associated information viewed through any standard web browser by appending the LSID to the prefix "http://zoobank.org/". The LSID for this publication is: urn:lsid:zoobank.org:pub:EA2A716F-4317-477A-B74E-B3DD25A6714B. The electronic edition of this work was published in a journal with an ISSN, and has been archived and is available from the following digital repositories: PubMed Central, LOCKSS.

## The Granton Shrimp Bed—Geological Setting

Within the Midland Valley of Scotland, a graben lying between the Highland Boundary Fault and the Southern Upland Fault, there are substantial thicknesses of Carboniferous sedimentary rocks. There was also much volcanic activity during the deposition of the sediments, episodic throughout the Carboniferous, which gives rise to a dramatic landscape. During the Dinantian there were complex and shifting patterns of sedimentation, influenced by the northward drift of the plate bearing what is now Scotland. Initially, in the eastern part of the Midland Valley, sedimentation was fluviatile and lacustrine, with thin coals; these sediments belong to the Tournaisian Inverclyde Group. But as the plate moved into a region dominated by tropical rainfall, during the early Visean (Strathclyde Group), a substantial lake formed, known as Lake Cadell. This was hemmed in to the north-west by the Clyde Plateau lavas, to the south-east by the Lower Palaeozoic Southern Uplands, to the northeast by an old Siluro-Devonian volcanic region and to the east by a great delta spilling down from a vast river system flowing from the north. The lake is generally regarded as having contained fresh or brackish water [19] although it was subject to intermittent invasion by the sea. Lake level fluctuated periodically, and oil shales formed from time to time. There are eleven main seams, formerly mined, from the 1870s to 1962. The Scottish Carboniferous, especially the oil shales, is renowned for the superbly preserved fish and ostracodes that lived in the lake, and the fossil plants and tetrapods from the surrounding forests. Yet there are horizons also where very delicate animals are found, of a kind not normally preserved. There are two of these Fossil-Lagerstätten, in which the most numerous fossils are ‘shrimps’, eumalacostracan crustaceans similar to those living today, though lacking claws and generally less modified. These are the Gullane and Granton (or Muirhouse) Shrimp Beds.

The Gullane Shrimp Bed, exposed at Cheese Bay, on the East Lothian coast and only 10 cm thick, was apparently deposited in a thermally stratified lake, probably a fresh-water satellite of Lake Cadell. It yields only the exquisitely preserved shrimp *Tealliocaris* Peach, 1908, and rare fish and amphibians [3, 20, 21]. Two poorly preserved specimens of analyzed here fossils come from this locality.

The Granton or Muirhouse Shrimp Beds are exposed on the southern shore of the Firth of Forth, about 3 km from the centre of Edinburgh. Here the sandstones of an abandoned delta lobe are overlain by lagoonal mud-shales, deposited in a relatively stagnant environment. The Granton Shrimp Bed is a 35–45 cm thick horizon within this sequence, consisting of alternating organic-rich and organic-poor dolostone laminae. These were interpreted [22, 23] as deposited in periodically exposed mud flats, and there is evidence of algal mats within this sequence also [5], some of which are distorted or enrolled through slumping or current activity. This sequence was presumably deposited at the margins of Lake Cadell or a satellite water body. It is generally regarded as having been laid down in a fresh or brackish lake, though subject to occasional marine incursions from the east.

## Systematic Paleontology

Remarks: The systematic position of the material described here is uncertain, we tentatively assign it to Hydrozoa. Detailed discussion on its systematic position is given below. Systematics here follow Schuchert [9].

Phylum: Cnidaria Verill, 1865 (?)
Class: Hydrozoa Owen, 1843 (?)
Subclass: Hydroidolina Collins & Marques, 2004 (?)
Order: Anthoathecata Cornelius, 1992 (?)
Suborder: Capitata Kühn, 1913 (?)
Family: Solanderiidae Marshall, 1892 (?)
Genus: *Caledonicratis* gen. nov.
LSID urn:lsid:zoobank.org:act:75B03B4E-9193-4ED9-BB45-F55A6382130A

Derivatio nominis: From Latin *Caledonia–*Scotland and *cratis–*network, because of colony wall structure. The genus name is feminine.

Diagnosis: Colonies small, erect, arborescent, irregularly branched with predominant monopodial and dichotomous growth. They bud in one plane. Wall microstructure composed of evenly spaced, linear fibers, running parallel to the axis of stems, connected by transverse bars.

Remarks: The record of Carboniferous soft-bodied hydroids is restricted, with two species described from Mazon Creek (Westphalian, Illinois, USA)–the colonial *Drevotella proteana* Nitecki and Richardson, 1972 [24] of unknown affinities and the solitary *Mazohydra* (Schram and Nitecki 1975 [25]; Baliński et al. [14] consider it as a problematic hydrozoan). *Drevotella* has partially hydrorhizal growth, not observed on our specimens, its hydranths are broad and rounded, and the colonies are much larger than those of *Caledonicratis*, attaining several centimeters. *Caledonicratis* also differs from the Recent *Solanderia*, with the latter having larger colonies and bubble shaped gonophores.

**Fig 2:**
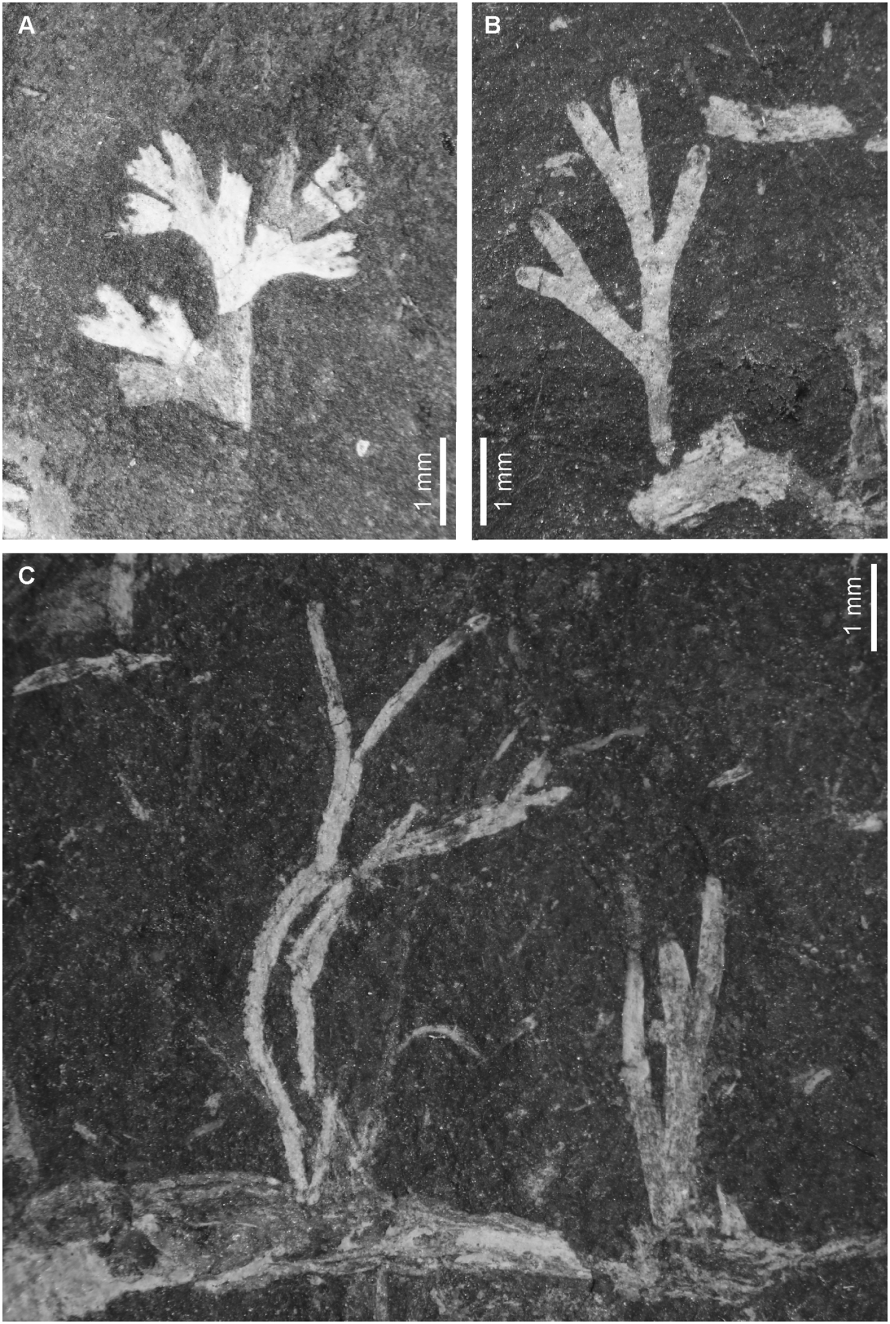
Examples of *Caledonicratis caridum* gen et sp. nov. (A) Specimen NMS.G.2013.34.20. (B) Specimen NMS.G.2013.34.1. (C) Specimen NMS.G.2013.34.26, encrusting a crustacean skeleton. All specimens from Granton, Edinburgh, Granton Shrimp Bed, Visean.

**Fig 3:**
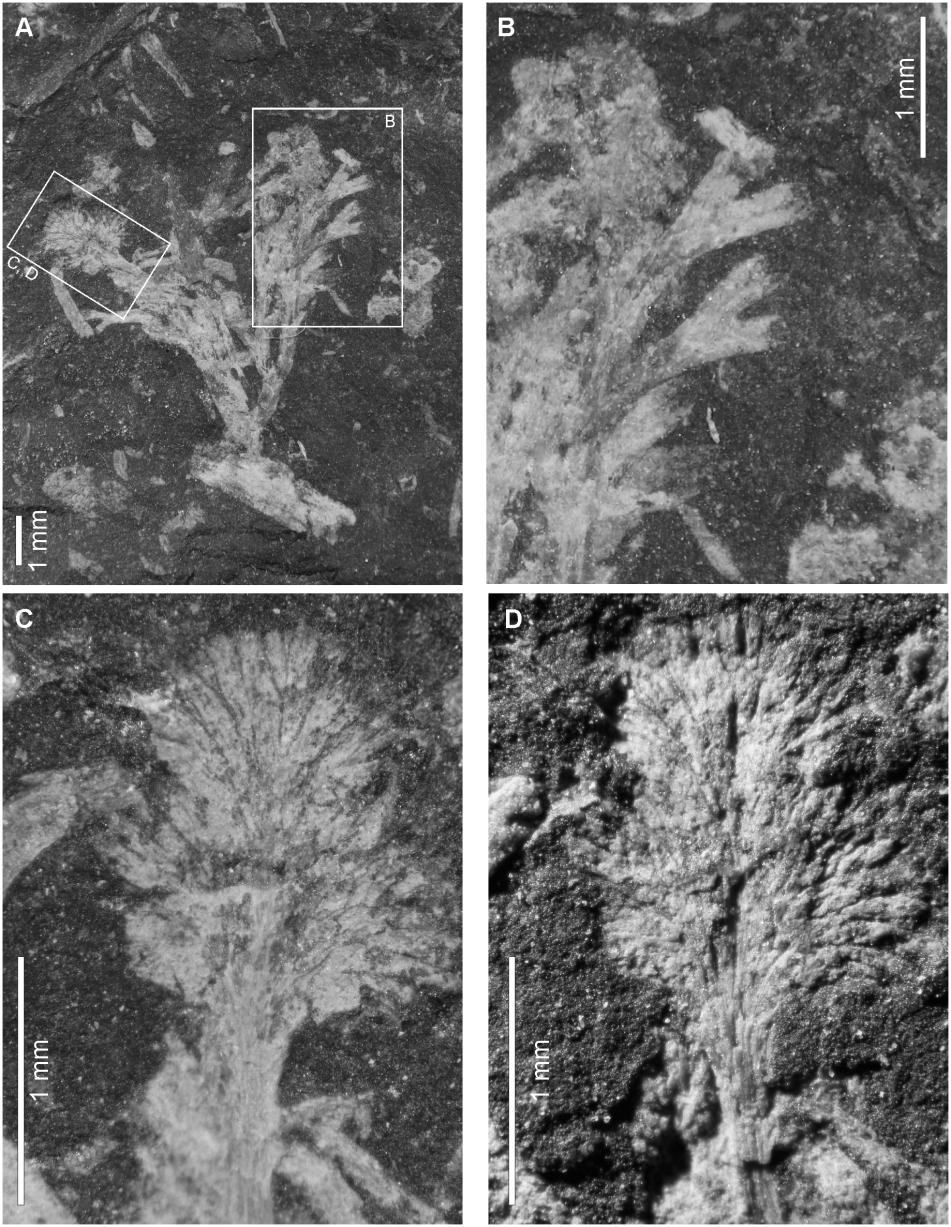
*Caledonicratis caridum* gen et sp. nov. with fan-like structures. (A) General view of the specimen. (B) Enlargement of a branch detail (C) and (D) is a fan-like structure interpreted here as gonophore. (C) Top lighting. (D) The same structure, acute angle lighting. Specimen RSM 2013.34.13 from Granton, Edinburgh, Granton Shrimp Bed, Visean.

**Fig 4:**
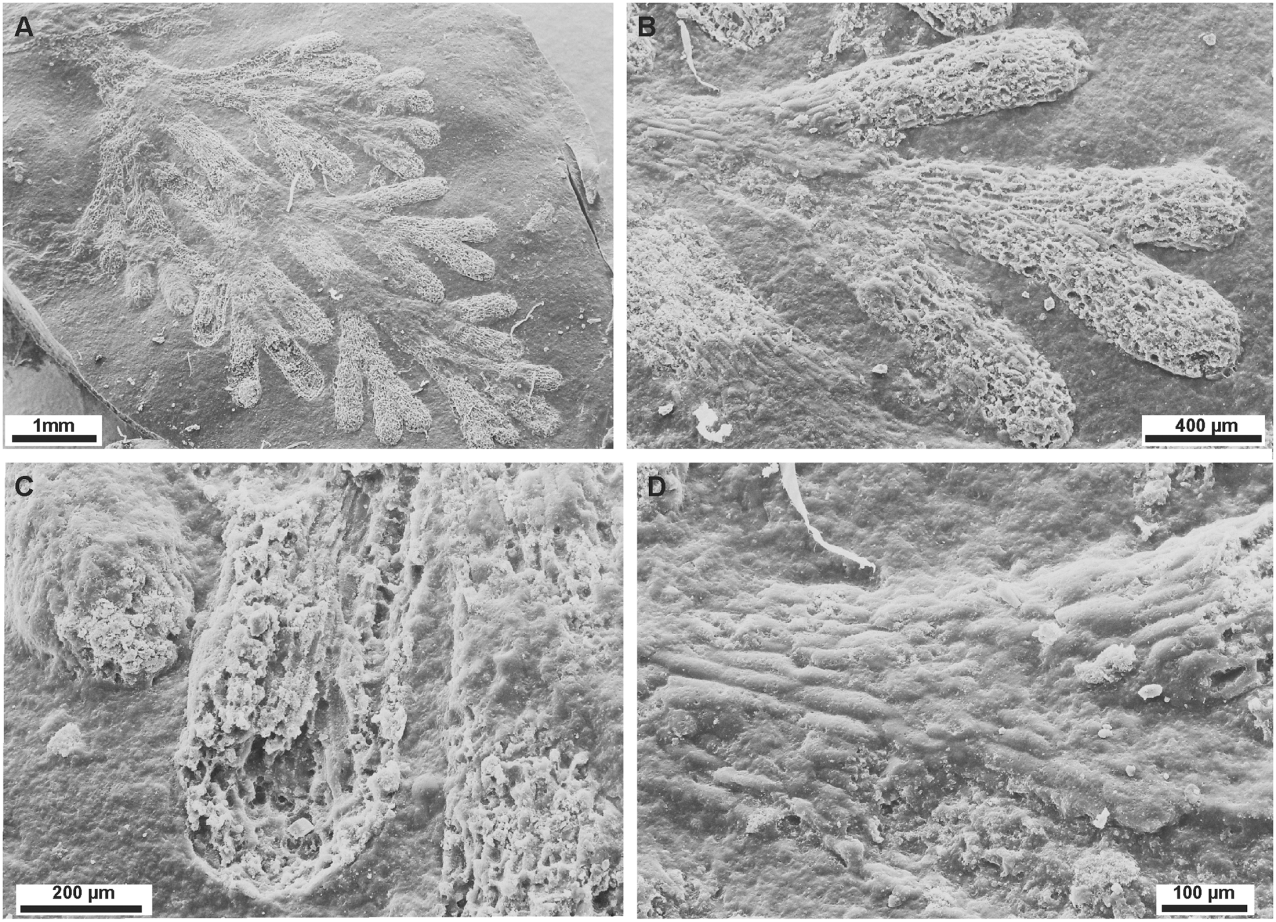
Microstructure of *Caledonicratis caridum* gen et sp. nov., holotype. (A) General view of a branch. (B-D) Details of the microstructure composed of evenly spaced, linear fibers, running parallel to the axis of the stems. SEM images of the holotype, specimen NMS.G.2015.1.1 from Granton, Edinburgh, Granton Shrimp Bed, Visean.

**Fig 5:**
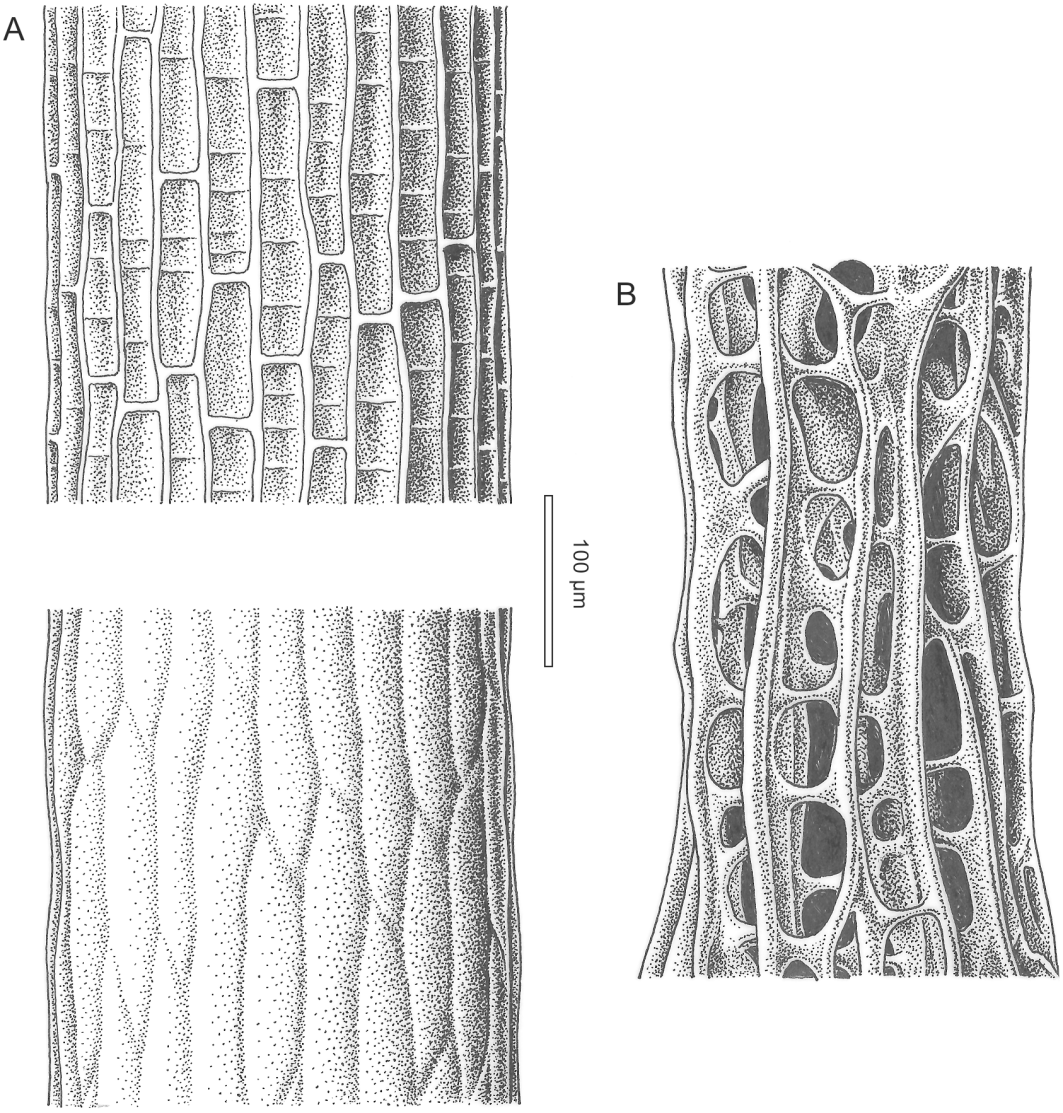
Comparison of *Caledonicratis caridum* gen. et sp. nov. and extant *Solanderia minima*. (A) A schematic drawing of a *C*. *caridum* branch, based on the holotype. Upper picture corresponds to the microstructure shown on the Fig 4B and 4C, lower corresponds to structures shown on the Fig 4D. Note that structure visible on the lower picture may be a diagenetic coating. (B) Microstructure of *Solanderia minima* (picture on the basis of the pl. V fig. 3 from [44]). Drawings by Bogusław Waksmundzki.

*Caledonicratis caridum* sp. nov.
LSID urn:lsid:zoobank.org:act:9565CF21-1292-467C-9701-848B491B22B9
Figs 2, 3, 4 and 5

1983. *Branching organisms*. Briggs and Clarkson, p. 164, pl. 20, figs 13–17.

1991. *Hydroid*. Briggs et al. 1991, text-fig. 12b.

Diagnosis: as for the genus

Material:

Holotype: Specimen NMS.G.2015.1.1 from Granton.
Paratypes: Granton: RSM.1982.66.18, RSM.1982.66.19, RSM.1982.66.21, RSM.1984.46.15, NMS.G.2013.1 to 13, 15 to 22, 23, 25 to 31; Cheese Bay: RSM.1984.46.11 RSM.1984.46.12

Derivatio nominis: *caridum*, latinized plural genitive of Greek *caris–*shrimp, because found in Granton Shrimp Beds.

Stratum typicum: Granton Shrimp Beds, Gullane Formation, Strathclyde Group, Visean.

Locus typicus: Granton, Edinburgh, UK.

### Description

#### The colony

Colonies small, erect, arborescent, reaching maximally 10 mm in length. Branching may be very irregular, but the general pattern can be described as monopodial with secondary dichotomous outgrowths. The colonies are preserved lying on bedding planes, and therefore are flattened. There is negligible overlap between branches even in extensively ramifying branches, and this indicates that the branching is essentially in one plane ([5]: p. 165). In some of the specimens the main growing tip gives rise to lateral branches, which give rise to further, often bifurcating branches. The angle between them usually ranges between 20 and 30°.

Branches are elongated, sometimes slightly thickened at the proximal end. They are usually about 0.5 mm wide, but there is large variation in our material—some are shorter and wider, some are long and slender. At the, or near to the proximal end of a branch, a small black dot often occurs ("small nodule of dark mineral"; [5]), which is difficult to interpret. This structure does not occur on all specimens and is not visible on the specimen with preserved microstructures. In some specimens there is a dark line running along the branch, close to its middle.

#### Fan-like structures

Besides dendroidal colonies, several isolated fan-like structures have been found (Fig 3; specimens NMS.G 2013.34.2 NMS.G 2013.34.7, NMS.G 2013.34.8, NMS.G 2013.34.13). These structures seem to be composed of narrow tubes, with whitish thin edges and a dark, nearly black, thick axial structure. Their size ranges from 1.5 to 4.5 mm. On one of the specimens (NMS.G 2013.34.13) this structure seems to be connected with the *Caledonicratis caridum* colony and it seems to have continuous structures with the rest of colony (Fig 3).

#### Microstructure

On one specimen (NMSG.2015.1.1) the microstructure of the fibers is well preserved (Fig 4). The structure is composed of evenly spaced, linear fibers, running parallel to the axis of stems. Some of these fibers reach width of 20 μm, but they may have been thickened during diagenesis. They are spaced 20–30 μm apart. These linear fibers are connected by transverse bars at intervals of 50–120 μm. It seems that these structures form the walls of a hollow tube. At the proximal part of the specimen under discussion this microstructure seems to be covered by another layer (Fig 4B—top left corner and Fig 4D, see reconstruction on Fig 5). The exact spatial relation between this layer and the fibrous one is unclear. This second layer may be either primary or diagenetic. Fibrous microstructure is faintly visible on two other specimens (NMS.G 2013.34.4 and NMS.G 2013.34.13).

### Biological affinities of the Granton fossils

Briggs and Clarkson [5] briefly described and illustrated the branching organisms from the Granton Shrimp Bed. They discussed the possible biological affinities of these fossils and stated that they may be either bryozoans, hydrozoans or algae. We briefly summarize the arguments *pro* and *contra* for various possible affinities, adding also poriferans.

Our material slightly resembles biocalcifying Devonian and Carboniferous algae, such as for example the dasycladacean *Issinella* [26]. Such algae are usually an order of magnitude smaller (usually sub-millimetric sizes), besides, our specimens are preserved as phosphates rather than carbonates (see discussion on taphonomy); biocalcifying algae would be probably be preserved as the latter. On the other hand, specimens from Granton resemble strongly multicellular calcifying algae such as *Ungdarella* or *Komia*. They are most commonly regarded as algae [27, 28], however, their taxonomic status was often questioned and they have also been compared with stromatoporoids or hydrozoans [27, 29]. Both genera display elongated, rectangular cells not visible in any of our specimens; also the pattern of branching seems to be much less regular than that of the material investigated here ([30]: fig. 76). What is more, both genera are known from the late Palaeozoic shelf carbonates (they contributed to reef formation [31]), and therefore their presence in the Late Palaeozoic fresh or brackish water (see discussion on ecology) may be unlikely. Our material also resembles the non-calcifying alga *Perissothallus*, described from the late Carboniferous and Permian of USA and Germany. *Perissothallus* is similar in its branching pattern, but this alga is much larger, with immature thalli exceeding several cm, and reaching nearly 20 cm, but these algae are preserved as brown impressions [32]. It must be noted that the *Perissothallus* from USA has been found in similar paleoecological setting, that is freshwater with possible marine incursions [32]. Most of non-calcifying algae are preserved by carbonization, but in Phaeophyta cells contain calcium alginate, which might possibly lead to phosphate preservation. Therefore algal affinity cannot be definitely ruled out (such a conclusion has been already formulated by Briggs and Clarkson, [5]).

The reticular microstructure of our specimens may slightly resemble either demospongean sponges of the order Agelasida or some calcareous sponges. Representatives of the former order usually have characteristic monactine megascleres [33] invisible in our material. In the geological setting where calcareous fossils occur (such as mollusks: [5]) calcareous sponges would be preserved as calcite or aragonite rather than phosphates (see section on taphonomy).

Our specimens resemble also ctenostome bryozoans related to the genus *Alcyonidium* Lamoroux, 1813, from which they differ in their smaller size and budding in one plane. Also, the internal structure and arrangement of zooids within the colony branch of *Alcyonidium* is chaotic ([34]: fig. 1B); in our material such internal structures cannot be traced in the branches. Another ctenostome that slightly resembles our material is *Vesicularia* Thompson, 1830. Representatives of this genus are strongly chitinized, with dichotomous or trichotomous branching, but presence of elongated, bubble shaped autozooids [35, 36] make it different from *Caledonicratis*, where such structures are absent.

In the phylactolaematan *Plumatella* the zooids are much more distinct [37, 38], as is the pattern of branching, which in *Plumatella* is rarely dichotomous and moreover, the orifices of the zooids are usually on one side [38]. Moreover, the microstructure of the chitinous skeleton in *Plumatella* is solid [39], thus entirely different from the microstructure of *Caledonicratis*.

Most of our specimens do not have preserved skeletal microstructures, but one of them has a well preserved reticular, anostomosing structure as described above. Moreover, in several specimens remains of such structures are also present, but poorly visible. Chitinous internal skeletons composed of fibers are characteristic for the extant hydrozoan family Solanderiidae Marshall, 1892 [40, 41, 42, 43, 44]. As only one specimen has such structures well preserved, and on several others they are just faintly visible (as longitudinal ribs) it remains unclear how far these structures are representative for the whole material. As there is a morphologic continuum between the specimens, we assign them all to one species.

The family Solanderiidae is monogeneric, with *Solanderia* Duchassaing & Michelin, 1846, being the only genus. This genus contains probably seven extant species [9, 41]. Molecular analyses show that representatives of this family are closely related to *Millepora*, *Zanclea* and *Asyncoryne* [11]. The chitinous skeletons of *S*. *minima* (Hickson, 1903) or *S*. *secunda* (Inaba, 1892) resemble structures visible in our material (cf. [45]: pl. 5, figs. 1–4 and pl. 12, figs. 1–4). Comparison of skeletal structures of *Caledonicratis* and *Solanderia* is shown on the Fig 5. Ridges seen in *Caledonicratis* are not exactly the same as in *Solanderia*, but one must remember that images of *Solanderia* are based on extant specimens, and the material of *Caledonicratis* is flattened and remineralized. There is also significant variation in the skeleton shape of *Solanderia*, both intraspecifically and intragenerically [45]. Also the budding in one plane is characteristic of several species of *Solanderia* [40], especially in small colonies [42]. Although solanderiids usually form larger colonies, such species as *S*. *secunda* may be as large as 4–8 cm in height [46], or *S dendritica* attaining 7 cm in height [45] thus sizes very similar to these of *Caledonicratis*.

The fan-like structures that are described above may be interpreted as gonophores. They are different from gonophores known from the recent *Solanderia*; in the latter genus the fixed sporosacs [45] are round [43, 45, 47], but they may also show distinct traces of radial canals [30, 43, 48]. The size of *Solanderia* gonophores is less than that of the structures discussed here. Male gonophores of *Solanderia secunda* have 200–250 μm in length, and female reach 450 μm [43]. In these species where gonophores are known, they are placed on short stalk, having 1/3 of the gonophore length [30]. It also must be kept in mind that our material is flattened, therefore obtaining its original shape requires restoration. It may be assumed that the observed shape is close to a hypothetical longitudinal section of this structure, therefore similar to a slightly conical ellipsoid. Another possible interpretation of these structures is that they may also resemble hydranths of *Cladocoryne*, where tentacles form a fan-shaped structure; both are similar in size [49].

It is difficult to interpret the black mineral grouping at the end of branches, especially since it does not occur in all specimens. These may be either thorns, which occur in some solanderiids (e. g. [45]), however this interpretation is doubtful, as they do not occur regularly. Another interpretation may be that at these places occur structures other than normal hydranths (such as attachment places of gonophores) that had a slightly different chemical composition, causing black mineral grouping. On the other hand, if interpreted as algae, these black mineral groupings might represent reproductive conceptacles [5]. The origin of these structures is, however, speculative.

Taking into account: microstructure of probably chitinous origin, branching pattern and possible polymorphism of zooids we may state that our material is either related to the Recent family Solanderiidae, which seems to be more probable, or to an alga similar to *Perissodactylus*.

## Ecology of *Caledonicratis caridum* gen et sp. nov.

The fauna in the Granton Shrimp Beds is much more diverse than that of the Gullane Shrimp Beds. Most of delicate fossils occur in bands, very probably as a result of mass mortality events. The commonest fossils are crustaceans, the small, ubiquitous and endemic *Waterstonella*, the larger, rarer predator *Anthracophausia*, uncommon *Anthracocaris*, *Tealliocaris* and *Pseudogalathea*, and very rare *Bairdops* [4, 5, 50]. There are also a number of individuals of the syncarid *Minicaris*, and the large ostracode *Eocypridina*. The salinity of the original habitat in which these lived remains uncertain, since both fish and crustaceans abound today in marine to freshwater environments. Living syncarids, however, are known only from fresh-water lakes and rivers in Tasmania. The diversity of crustaceans testifies to a dramatic Lower Carboniferous eumalacostracan radiation. There are also unequivocal marine fossils in the Granton Shrimp Bed, orthocone cephalopods, the marine planktonic worm *Eotomopteris* [51], and several conodont animals [1, 52, 53, 54]. The salinity habitat of the chordate *Conopiscus* is unknown [55]. A likely isopod has recently been discovered, along with a new *Eotomopteris* specimen [56], and a possible sipunculid has been recorded [6].

As briefly outlined, fossil faunas of the Granton Shrimp Beds give contradictory information on the salinity of the *Caledonicratis* habitat. A likely scenario, which reconciles all the apparently conflicting lines of evidence, is that Lake Cadell normally contained fresh or brackish water. The endemic fauna consisted of the abundant ‘shrimps’ *Waterstonella* and *Anthracophausia*, living in the lake along with fish and probably other crustaceans. The syncarids may also have lived in the lake itself or were inhabitants of rivers draining into the lake. Periodically there were storm surges from the east, and during such times marine organisms were brought in. The conodont animals are sometimes partially rotted, as is the case also with the tomopterids, thereby suggesting long-distance transport. So, in what conditions did the *Caledonicratis* organisms originally live? They are very abundant, as are the crustaceans, and occur at several levels, including all those where crustaceans are found. In some instances hydroids are actually growing on the bodies or exuviae of the crustaceans (Fig 2A). This indicates that they lived in the same habitat. It is likely, therefore that *Caledoniocratis* organisms were of fresh or brackish water origin; such is the most parsimonious interpretation. This conflicts with the data on modern solanderiids, which are exclusively marine [9]. As stated above, these organisms may be related to brown algae, which are also nearly exclusively marine [57]. Fresh or brackish water organisms have usually efficient osmoregulatory mechanisms and high tolerance to salinity changes [58]. Although very rarely, sometimes members of a single hydrozoan genus can inhabit brackish and fully marine environments, as it is in case of *Pachycordyle* (Anthoathecata: Filifera; [59]). While this is only distantly related to solanderiids, it shows that shifts between environments are possible in hydrozoans. It might therefore have happened that during the long evolution of this group, some members of the Solanderiidae colonized brackish environments.

Recent hydrozoans often encrust crustaceans, mostly crabs and hermit crabs (e.g. [60, 61, 62, 63]). Shrimps rarely host hydrozoans, as is in the case of *Earleria corachloeae* [64]. As representatives of *Caledonicratis caridum* sometimes occur on shrimps (or their exuviae) it may be inferred that (at least in cases of *syn vivo* encrustation, which cannot be proven in this case) they were ecologically similar to the case of *E*. *corachloeae*.

Colonies discussed here are either found isolated, or attached to shrimp exuviae or carcasses. It is unknown whether the attachment took place *syn vivo* or *post mortem*, using carapaces only as hard substrate. If the hydrozoans were attached to living shrimps, the relationship between the two might have been close to parasitic, similar to extant filiferans *Hydrichthys* Fewkes, 1887 or *Larsonia* Boero, Bouillon et Gravilli 1991 [65]. These are not closely related to Solanderiidae, but the bryozoan symbiont *Zanclella*, possessing either few or no tentacles [66, 67] may be considered as similar to our material. Unfortunately, without further material this issue cannot be resolved.

### Taphonomy

The specimens are preserved on bedding planes, therefore flattened. However, the negligable overlap of branches suggests that the budding in the colonies under discussion was in a single plane.

**Fig 6:**
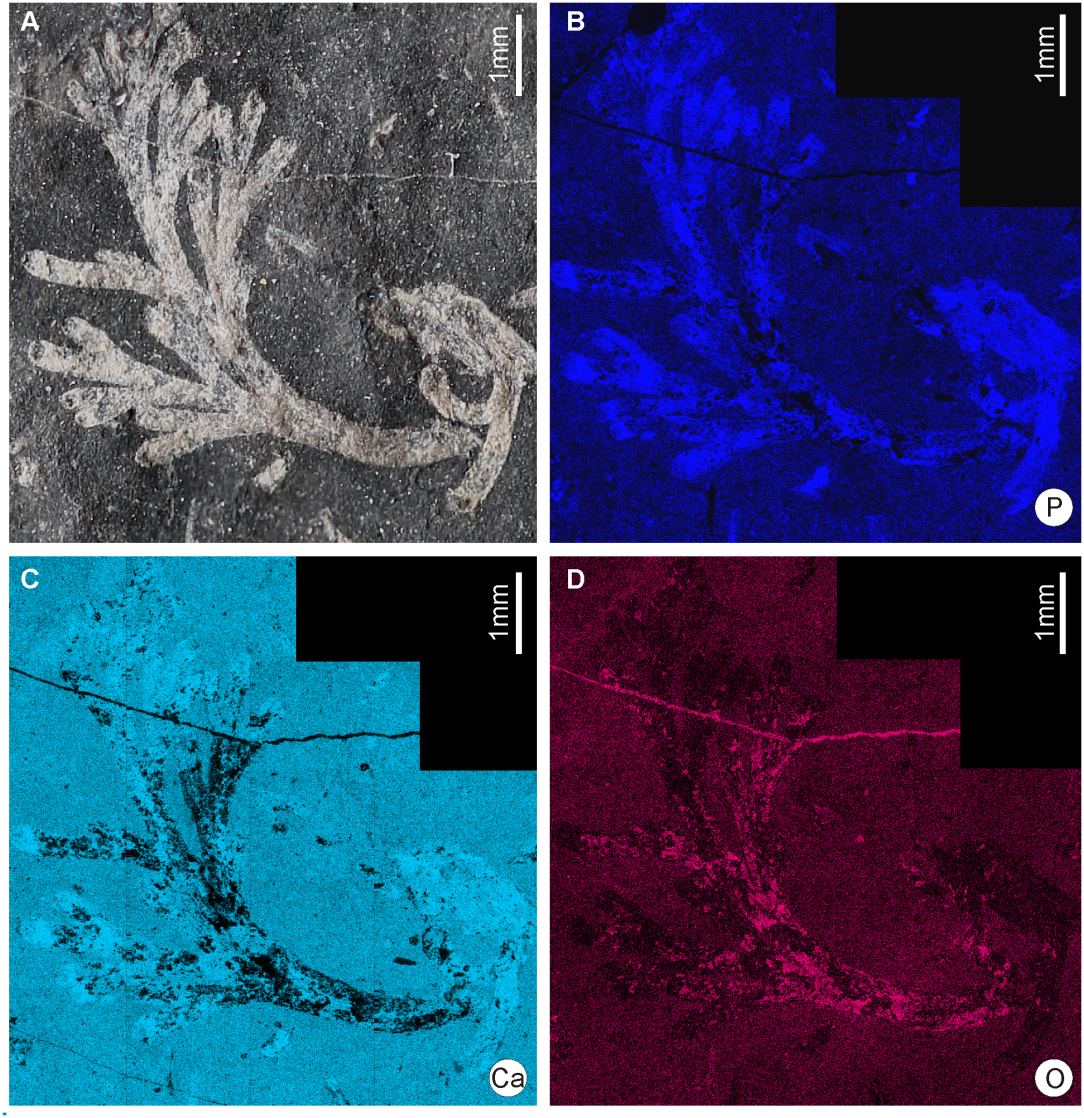
EDS elemental mapping of *Caledonicratis caridum* gen et sp. nov. (A) General view of a specimen. (B) Phosphorus concentrations. (C) Calcium concentrations. (D) Oxygen concentrations. The lighter the color, the higher relative abundance of a given element. Please note that abundances are not comparable between pictures, and reference level is different for each element. Specimen RSM 1982.66.18.(P) from Granton, Edinburgh, Granton Shrimp Bed, Visean.

SEM/EDS analyses performed on three specimens (NMS.G.2014.34.13. NMS.G.2014.34.17 and RSM 1982-66-18) evidence the presence of phosphorus, calcium and oxygen (Fig 6). On these pictures, the relative abundance of a given element is shown—the lighter the colour, the higher the relative abundance of a given element. Such concentrations evidence phosphatization of the material [68]. Phosphorus is present along nearly the whole outline of the colony, but absent in the central part of the specimen (Fig 6b), but as specimens are partly preserved on the part, and partly on the counterpart it can be assumed that fragments of phosphatized elements were left on the counterpart. The area around the branches of the fossil body has a very low concentration of phosphorus. On the other hand, absence of microstructures in most of the specimens may suggest that they were non-mineralized. These observations may therefore suggest that the skeleton of *Caledonicratis* was built by a substance possibly related to chitin [69], as it is in modern solanderiids. The very early phosphate diagenesis may be either related to a contemporaneous algal bloom, and deposited as fluorapatite by bacteria which covered and penetrated the dead bodies of the various invertebrates retaining the form of the animal after the soft parts had rotted away [4]. Another explanation, mentioned above is that brown algae are possibly more prone to phosphatization, therefore this way of phosphatization may suggest algal affinity. These phosphates may be also of secondary origin, as a result of later phosphatization of carbonates. However, also shrimps are preserved in similar way [4] and this suggests that hydroid skeletons were originally chitinous. It can also be added that in these cases where chitinous skeletons are preserved as phosphates, algae are preserved as carbonaceous films, as is in the Famennian of Kowala, Holy Cross Mountains, Poland [68].

The calcium distribution is somewhat different from that of phosphorus. This may be caused by presence of other minerals, such as calcite. Also the oxygen distribution pattern may be affected by presence of other minerals, as this element occurs commonly in e. g. dolomite (the bulk of rock in the specimens is dolomitic).

## Possible Evolutionary Significance of *Caledonicratis caridum* gen et sp. nov.

Assuming the hydrozoan nature of *Caledonicratis caridum* gen. et sp. nov. and its possible relation to the extant family Solanderiidae, its discovery may be significant in understanding of the evolution of Zancleida. As already shown [11], the Solanderiidae are one of the most derived families within the Zancleida. The finding of a possible solanderiid from the Early Carboniferous indicates that the divergence of all families of the Zancleida probably had taken place not later than in the Visean.

If the new species described here is an alga, its evolutionary significance would depend on more precise classification, which on present material is not possible.

## Conclusions

1. *Caledonicratis caridum* gen et sp. nov. is probably a hydrozoan, showing potential affinities with the extant family Solanderiidae Marshall 1892; it therefore may suggest the fossil record of Solanderiidae in the Visean. Its algal affinity, although possible is much less likely.
2. *Caledonicratis caridum* gen et sp. nov. lived in the freshwater or slightly brackish Visean Lake Cadell; it therefore seems that Solanderiidae might have been primarily freshwater or brackish, whereas modern representatives of this family are exclusively marine.
3. The Solanderiidae are the most derived family within the Zancleida. If *Caledonicratis* is indeed a solanderiid it can be concluded that the divergence of all families of Zancleida probably had taken place not later than in the Visean.
