# Correction: Enigmatic Fossils from the Lower Carboniferous Shrimp Bed, Granton, Scotland

**DOI:** 10.1371/journal.pone.0150047

**Published:** 2016-02-19

**Authors:** Mikołaj K. Zapalski, Euan N. K. Clarkson

The following text, which appears as the second paragraph of the Introduction, should not be included in the Introduction, but instead in the Fig 1 legend: “LWM and HWM refer to Low Water Mark and High Water Mark respectively. Based upon British Geological Survey geological map. 1:50000 Series. Edinburgh Scotland Sheet 32 E [7], with the permission of the British Geological Survey.” Please see the complete, correct Fig 1 and its legend below.

**Fig 1:**
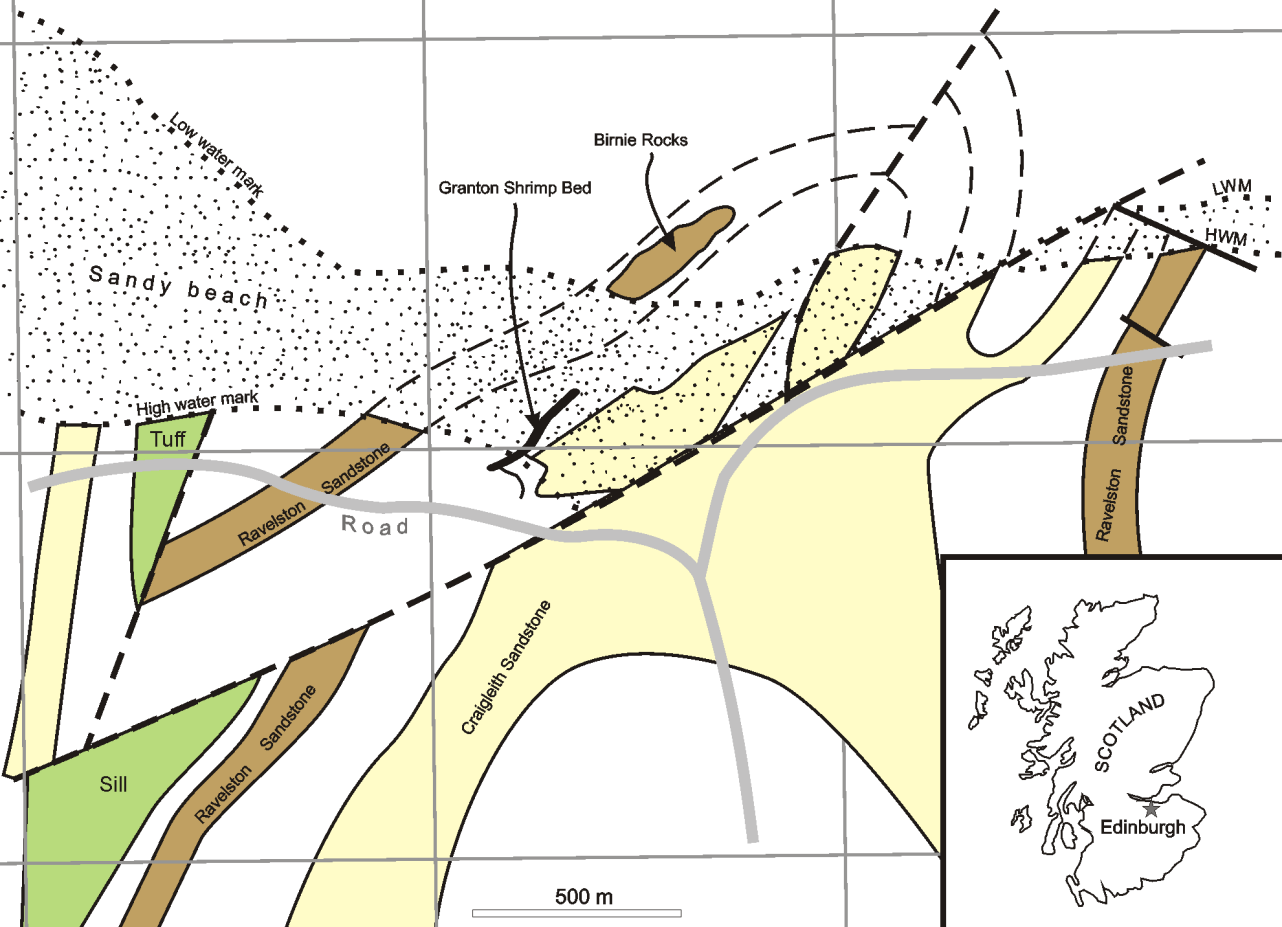
A map showing the locality of the Granton Shrimp Bed at Granton, Edinburgh. LWM and HWM refer to Low Water Mark and High Water Mark respectively. Based upon British Geological Survey geological map. 1:50000 Series. Edinburgh Scotland Sheet 32 E [7], with the permission of the British Geological Survey.
